# PLAU Promotes Cell Proliferation and Epithelial-Mesenchymal Transition in Head and Neck Squamous Cell Carcinoma

**DOI:** 10.3389/fgene.2021.651882

**Published:** 2021-05-20

**Authors:** Guangjin Chen, Jiwei Sun, Mengru Xie, Shaoling Yu, Qingming Tang, Lili Chen

**Affiliations:** ^1^Department of Stomatology, Union Hospital, Tongji Medical College, Huazhong University of Science and Technology, Wuhan, China; ^2^School of Stomatology, Tongji Medical College, Huazhong University of Science and Technology, Wuhan, China; ^3^Hubei Province Key Laboratory of Oral and Maxillofacial Development and Regeneration, Wuhan, China

**Keywords:** prognostic biomarker, EMT, PLAU, *TNFRSF12A*, head and neck squamous cell carcinoma

## Abstract

Plasminogen activator, urokinase (uPA) is a secreted serine protease whose Dysregulation is often accompanied by various cancers. However, the biological functions and potential mechanisms of PLAU in head and neck squamous cell carcinoma (HNSCC) remain undetermined. Here, the expression, prognosis, function, and coexpression genetic networks of PLAU in HNSCC were investigated by a series of public bioinformatics tools. A Higher PLAU level predicted a poorer clinical outcome. Meanwhile, functional network analysis implied that PLAU and associated genes mainly regulated cell-substrate adhesion, tissue migration, and extracellular matrix binding. The top 4 significantly associated genes are *C10orf55*, *ITGA5*, *SERPINE1*, and *TNFRSF12A*. Pathway enrichment analysis indicated that PLAU might activate the epithelial-to-mesenchymal transition (EMT) process, which could explain the poor prognosis in HNSCC. Besides, genes associated with PLAU were also enriched in EMT pathways. We further validated the bioinformatics analysis results by *in vivo* and *in vitro* experiments. Then, we found that much more PLAU was detected in HNSCC tissues, and the silencing of PLAU inhibit the proliferation, migration, and EMT process of CAL27 cell lines. Notably, the downregulation of PLAU decreased the expression of *TNFRSF12A*. Moreover, knockdown *TNFRSF12A* also inhibits cell proliferation and migration. *In vivo* experiment results indicated that PLAU inhibition could suppress tumor growth. Collectively, PLAU is necessary for tumor progression and can be a diagnostic and prognostic biomarker in HNSCC.

## Introduction

Head and neck cancer derives from the oral cavity, pharynx, and other upper aerodigestive tracts, in which HNSCC is the most common one (Mehanna et al., 2010). Carcinogenic factors of HNSCC contain smoking and alcohol as well as human papillomavirus (HPV) infection (Chow, 2020). Due to the high incidence of HNSCC and most HNCSS patients present a tendency of recurrence or distant metastasis, the prognosis of HNSCC patients is poor (Argiris et al., 2008). Certain mechanisms and pathways for tumor formation have been identified for targeted drug development, such as EGFR inhibitors. However, the benefit of existing drugs is dissatisfactory (Sacco and Cohen, 2015). Thus, the identification of new drug targets for the therapy and prognosis of HNSCC seems urgent.

*PLAU* encodes a secreted serine protease uPA (Mekkawy et al., 2014). uPA belongs to the plasminogen activator (PA) system and it serves to convert plasminogen to plasmin which is a broad-spectrum protease (Dano et al., 2005). Thus, uPA is involved in the basement membrane and extracellular matrix degradation process which counts for tumor invasion and metastasis (Mekkawy et al., 2014). Also, accumulating evidence indicated that the uPA system could participate in cell proliferation/apoptosis and EMT via other signaling pathways (Lester et al., 2007; Jo et al., 2009). Increasing *PLAU* expression was detected in various tumors, which is associated with patient survival, thus the uPA system could be a biomarker in malignancies (LeBeau et al., 2015; Su et al., 2016; Mahmood et al., 2018; Novak et al., 2019). However, the function of *PLAU* and specific mechanisms in HNSCC has not been determined till now.

We studied *PLAU* expression, mutation, and prognosis value in data from HNSCC patients with various open-access databases. We perform multi-dimensional analysis of genomic alteration and functional networks related to *PLAU* in HNSCC. Besides, we uncovered the biological function of PLAU in HNSCC through multiple experiments. Our results could confirm PLAU as a diagnosis and treatment target for HNSCC.

## Materials and Methods

### Data Mining

#### Oncomine

Oncomine^1^ is the largest cancer microarray database (Rhodes et al., 2004). We used Oncomine to analyze the mRNA levels of PLAU between HNSCC and adjacent normal tissues. We focused on several HNSCC studies, including Peng Head-Neck, Ginos Head-Neck, Estilo Head-Neck, Sengupta Head-Neck, Ye Head-Neck, Pyeon Multi-cancer (Ginos et al., 2004; Sengupta et al., 2006; Pyeon et al., 2007; Ye et al., 2008; Estilo et al., 2009; Peng et al., 2011).

#### UALCAN

UANLCAN^2^ is a web tool based on TCGA (The Cancer Genome Atlas) datasets (Chandrashekar et al., 2017). The analysis of PLAU expression in HNSCC and other cancer sub-groups including gender, ages, stages, or other features was conducted with UANLCAN. The survival probability was also analyzed by the UANLCAN database.

#### cBioPortal Analysis

The genetic profile of PLAU in HNSCC was obtained by cBioPortal^3^ (Gao et al., 2013). The Oncoprint module showed the genetic alteration of PLAU in HNSCC.

#### GEPIA

GEPIA^4^ is a customized cancer database (Tang et al., 2017). We used GEPIA to visualize the analysis of specific genes from HNSC samples.

#### LinkedOmics

linkedOmics^5^ integrates multi-omics data from TCGA, which contains three analysis modules (Vasaikar et al., 2018). The PLAU association result in HNSCC (*n* = 520) was explored by the LinkFinder tab which used Pearson’s correlation coefficient. Then we used Gene Set Enrichment Analysis (GSEA) in the LinkInterpreter module to obtain the analysis of GO, KEGG pathways enrichment. The rank criteria were *P*-value, minimum genes size was 3 and simulations was 500.

#### Timer

The expression of PLAU in different cancers and immune infiltration levels were assessed by Time (cistrome. shinyapps.io/timer) (Li et al., 2017).

#### GSCALite

We investigated the single nucleotide variation, pathway activity, and drug sensitivity of query genes with TCGA HNSC samples by GSCALite^6^ (Liu et al., 2018).

### Tissue Specimens

Five matched pairs of oral squamous cell carcinoma tissues and matched adjacent non-tumorous tissues were obtained from the Department of Stomatology, Huazhong University of Science and Technology affiliated Union Hospital. This study protocol was approved by the Institutional Research Ethics Committee of Tongji Medical College (Wuhan, China). These specimens were further used in immunohistochemistry staining.

### Animal Study

To establish subcutaneous xenograft, CAL-27 cells (1 × 10^7^) were subcutaneously injected into 10 male BALB/c nude mice. One week after injection, 2 groups (*n* = 5 per group) mice were randomly distributed. uPA inhibitor UK-371804 (Selleck, S8457, dissolved in DMSO, 20 μg per mouse, treatment group) or DMSO (1 μL per mouse, control group) was twice weekly intratumorally injected. Vernier calipers were used for measuring the tumor (twice a week). Two weeks later, the subcutaneous tumors were harvested to calculate the volume by using the formula (length × width^2^)/2.

### Cell Culture

The human OSCC cell line CAL27 used for experiments was obtained from ATCC. CAL 27 cell lines were cultured in DMEM (Hyclone) supplemented with 10% fetal bovine serum (FBS), 100 μg/mL streptomycin, and 100 U/mL penicillin (Beyotime), at 37°C in 5% CO_2_.

### Small Interfering RNA (siRNA) Transfection

Specific siRNA (GenePharma) and non-specific control were transfected into CAL27 cells with lipofectamine 3,000 (Invitrogen) reagents. After 4–6 h transfection, the medium was refreshed and the transfection effect was measured by real-time qPCR. The sequences of the siRNA are shown in Supplementary Table 1.

### Quantitative Real-Time PCR

RNA was isolated from cells using TRIZOL (TAKARA) according to the manufacturer’s instructions and reverse-transcribed using HiScript II Q RT SuperMix for qPCR (Vazyme). Then, quantitative PCR was performed by the StepOne Plus PCR machine (Life Technologies, Carlsbad, CA). The PCR conditions are listed as follow: 5 min at 95°C,then 15 s at 95°C, 30 s at 60°C for 40 cycles, 15 s at 95°C, 60 s at 60°C and 15 s ay 90°C. GAPDH was used as internal control and the relative mRNA levels were analyzed by the 2^–ΔΔ*CT*^ method. The primers sequences are showed in Supplementary Table 2.

### CCK-8 Assays

The proliferation and cytotoxicity of CAL-27 cells were measured by cell counting kit (CCK-8, Beyotime Institute of Biotechnology). In brief, CAL-27 cells were seeded into a 96-well plate (2,000 cells per well) and incubated for 48 h. After the CCK-8 reagent was added and incubated for 1 h, the optical density was detected at 450 nm wavelength. For cellular cytotoxicity assay, the CAL-27 cells were incubated with 0.34 nM bleomycin (Selleck, S1214) or 8.07 μM docetaxel (Selleck, S1148) for 48 h at 37°C.

### Wound Healing Assay

CAL27 cell lines were transferred to a 12-well plate and a linear wound was formed by scratching with 200 μL micropipette tip, then cultured for 24 h with serum-free medium. The cells were photographed at 0 and 24 h and the wound distance was used to assess the cell migration rate.

### Migration Assay

The migration capacity of CAL27 cells was evaluated using the transwell chambers (Corning Life Sciences, MA, United States). CAL27 cells (5 × 10^4^) with 200 μL serum-free medium were loaded into the upper chamber while medium supplemented with 10% serum were added to the lower well. After 24 h incubation, the top surface cells were removed and the rest were stained with crystal violet.

### Western Blot

The cells’ protein was collected by RIPA lysis buffer (Byotime, P0045), then the BCA protein assay kit (Biosharp, BL521A) normalizes the samples. The cell extracts were boiled with Sample Loading Buffer (Biosharp, BL529A) for 5 min. After separated by 10% SDS-PAGE gels and transferred to PVDF membrane (Millipore, Germany), the membranes were blocked in 5%BSA and incubated with primary antibodies at 4°C overnight. The primary antibodies are listed as follows: anti-PLAU (1:2,000, Proteintech, 17968-1-AP), anti-E-Cadherin (1:5,000, Proteintech, 20874-1-AP), anti-N-Cadherin (1:2,000, Proteintech, 22018-1-AP), anti-TWIST1 (1:1,000, Proteintech, 25465-1-AP), anti-SNAI1 (1:1,000, Proteintech, 13099-1-AP). Then the membranes were incubated with secondary antibody (Biosharp, BL003A) and the proteins were visualized by ECL enhanced chemiluminescence substrate (Millipore).

### Immunohistochemistry Staining

The oral cancer tissues and matched adjacent normal oral tissue were obtained from the Department of Stomatology, Huazhong University of Science and Technology affiliated Union Hospital. All the specimens were fixed with 10% buffered formalin, embedded in paraffin, and sectioned in sequence. The sections were deparaffinized by dimethyl benzene and boiled in alkaline Tris buffer for antigen repairing. UltraSensitiveTM SP IHC Kit (MXB, KIT-9710) was used to perform the immunostaining. After blocking, the slides were incubated with primary antibody for PLAU (1:200 dilution, Proteintech, 17968-1-AP) overnight at 4°C. After washing, the sections were immersed with biotin-conjugated IgG secondary antibody in IHC kit for 20 min, then with DAB for 2–5 min and stained with hematoxylin for 5 s. The sections were photographed with a Nikon microscope (Japan).

### Statistical Analysis

Statistical analysis was performed with GraphPad Prism 7.0 software and the significance was analyzed with Student’s *t*-test or ANOVA. In our study, *P* < 0.05 was considered statistically significant. All results were shown as Mean ± SD.

## Results

### PLAU Is Upregulated in Head and Neck Squamous Cell Carcinoma

**FIGURE 1:**
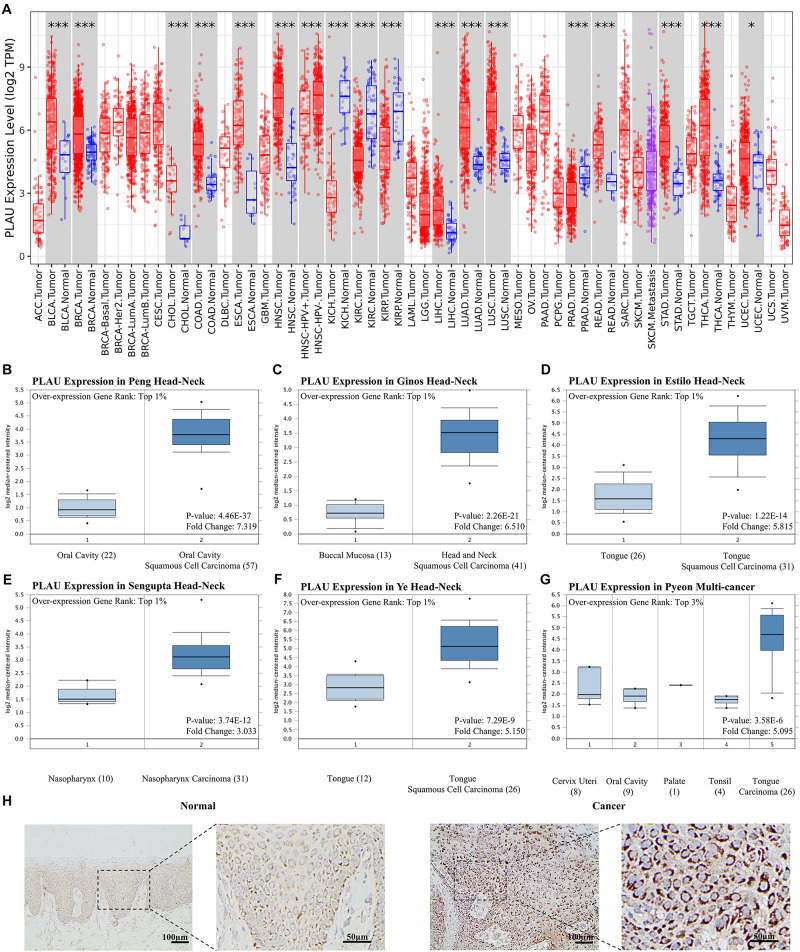
PLAU expression is upregulated in head and neck squamous cell carcinoma. **(A)** Relative *PLAU* expression levels in different cancers. **(B–G)** Boxplot showing *PLAU* mRNA levels in the Peng Head-Neck, Ginos Head-Neck, Estilo Head-Neck, Sengupta Head-Neck, Ye Head-Neck, Pyeon Multi-cancer, respectively. Levels of *PLAU* mRNA were significantly higher in tumor tissues than normal head and neck tissues. **(H)** Detection of PLAU expression levels in OSCC tissues and normal tissues with IHC.

**FIGURE 2:**
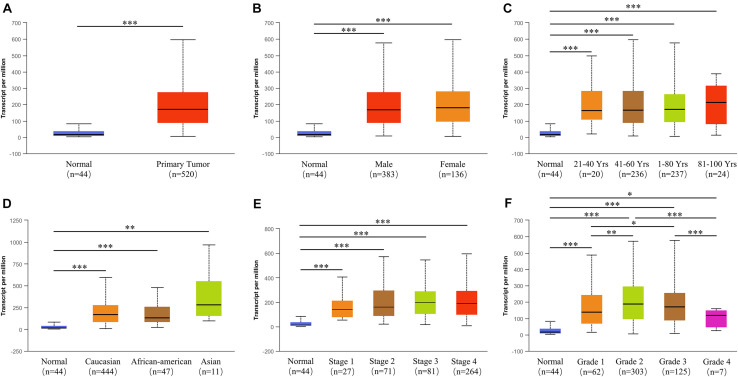
The relationship between *PLAU* expression and tumor subgroups in HNSCC. **(A)** Relative expression of *PLAU* in normal and HNSCC samples. **(B)** Relative expression of *PLAU* in normal individuals or HNSCC patients based on gender. **(C)** Relative expression of *PLAU* in normal individuals or HNSCC patients based on age. **(D)** Relative expression of *PLAU* in normal individuals or HNSCC patients based on race. **(E)** Relative expression of *PLAU* in normal individuals or HNSCC patients based on pathological stage. **(F)** Relative expression of PLAU in normal individuals or HNSCC patients based on tumor grade. *p < 0.05; **p < 0.01; ***p < 0.001.

To identify the role of *PLAU* as a pan-cancer biomarker, a comparison between tumor and normal tissues was completed and multiple types of cancers, such as BLCA and BRCA, showed a high level of *PLAU* in tumor tissues among TCGA samples (Figure 1A). Then we evaluated expression levels of *PLAU* between HNSCC samples and normal samples in various studies from Oncomine. *PLAU* transcription levels were significantly higher in HNSCC tumor tissues among different studies. All the fold differences reached beyond 3 (Figures 1B–G). Then, we used the immunohistochemistry staining to confirmed that the PLAU expression was higher in OSCC tissues than in adjacent normal tissues (Figure 1H). The relative quantitive analyzing results showed that the ratio of PLAU-positive area in adjacent normal tissues was 17.58% while that in OSCC tissues was 44.35%. Further analysis of HNSCC tissues from TCGA based on clinical and pathological information revealed a higher abundance of *PLAU* in primary tumor patients than healthy ones based on gender, age, ethnicity as well as tumor stages, and grades (Figures 2A–F). Especially, PLAU expression of grade 1 was lowest while PLAU expression of grade 2 was highest in HNSCC. Taken together, *PLAU* act as a crucial diagnostic biomarker in HNSCC.

### PLAU Is Negatively Correlated With HNSCC Prognosis

**FIGURE 3:**
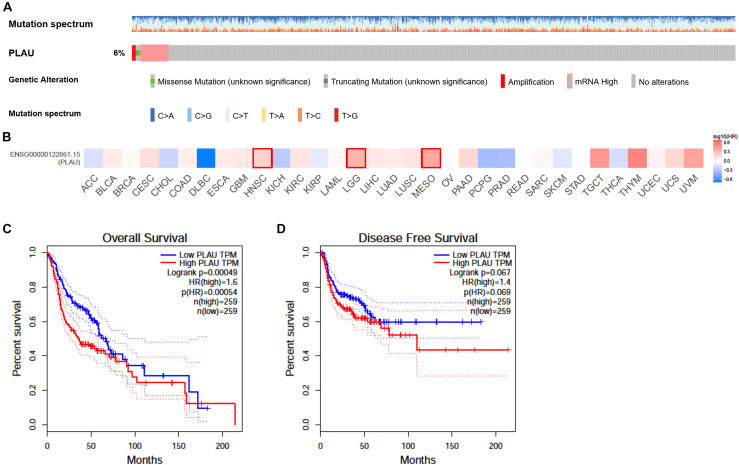
Alteration and prognostic value of *PLAU* in HNSCC. **(A)** The alterations of *PLAU* in HNSCC. **(B)** The association of PLAU expression and prognostic potential across different cancer types. **(C)** Overall survival of *PLAU* in HNSCC. **(D)** Disease-free survival of PLAU in HNSCC.

CBioPortal was chosen to determine the *PLAU* alterations in HNSCC based on TCGA database. *PLAU* was altered in about 6% of the total patients. Most of them belonged to mRNA upregulation, with amplification and missense mutation taking part in as well (Figure 3A). We also analyzed the survival with the help of GEPIA web tools. The survival analysis results of PLAU in multiple cancer types demonstrated that PLAU was only negatively correlated with HNSCC, Brain Lower Grade Glioma (LGG), and Mesothelioma (MESO) (Figure 3B). HNSCC patients with a low *PLAU* level showed a better clinical outcome compared to the high expression group in HNSCC patients. Bothe OS and DFS showed a consistent relationship with PLAU expression (Figures 3C,D). Therefore, it is convinced that PLAU could be a prognostic biomarker for terrible clinical outcomes.

### Enrichment Analysis of PLAU Associated Genes Network in HNSCC

**FIGURE 4:**
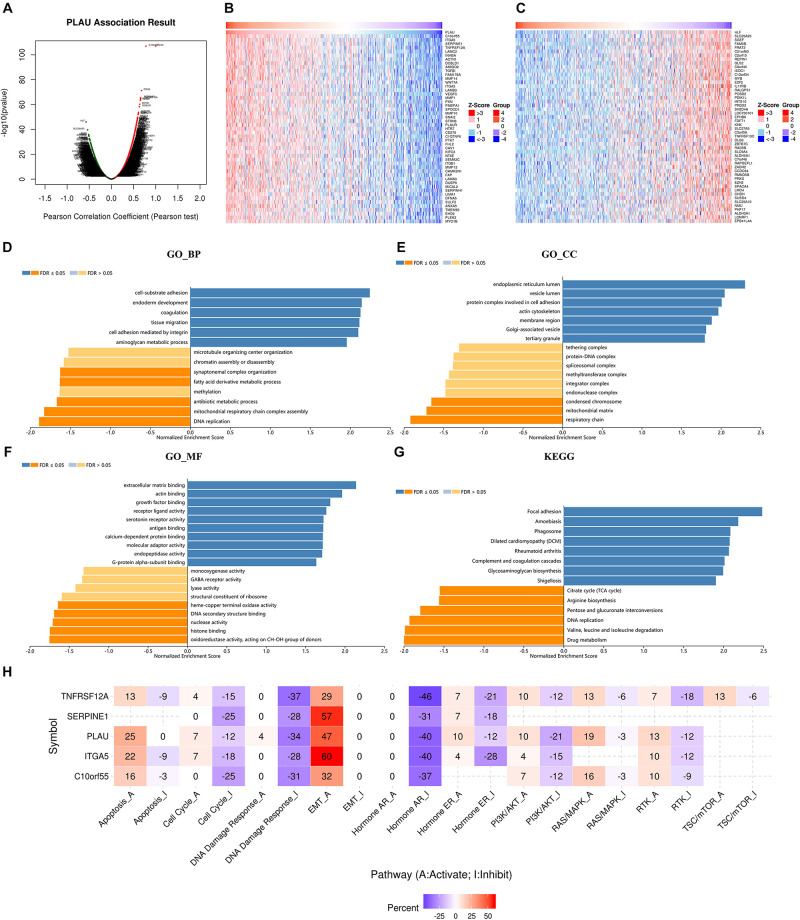
*PLAU* co-expression genes in HNSCC. **(A)** The genes correlated with *PLAU* were analyzed by Pearson test in HNSCC. **(B)** The top 50 genes positively correlated with PLAU in HNSCC. **(C)** The top 50 genes negatively correlated with PLAU in HNSCC. **(D–G)** Gene enrichment analysis of *PLAU*-associated genes in HNSCC. BP, biological process; CC, cellular components; MF, molecular functions. KEGG pathway analysis. **(H)** The relationships between hub genes expression and pathway activity groups.

We next used LinkedOmics to analyze RNA sequencing data of HNSCC samples. The volcano plot depicted that 4,993 genes showed positive correlations with *PLAU*, while 7,385 showed negative correlations with *PLAU* significantly (Figure 4A). The top 50 significantly positively and negatively correlated co-expression genes were presented by heatmaps (Figures 4B,C). Four most significantly correlated genes, *C10orf55* (cor = 0.7803, *P* = 1.133e-107), *ITGA5* (cor = 0.6805, *P* = 649e-72), *SERPINE1* (cor = 0.6592, *P* = 3.887e-66) and *TNFRSF12A* (cor = 0.6569, *P* = 1.554e-65), were selected according to the *P*-value and used to establish linear regression models (Supplementary Figure 1A). This result indicated that *PLAU* might function along with a large group of genes during HNSCC progress.

Significant GO term by LinkedOmics revealed that *PLAU* along with its co-expressed genes were mainly enriched in cell-substrate adhesion and extracellular matrix binding, which indicated abnormality of cell membrane proteins and were correlated to the EMT process. This just coincided with the result that the most enriched cellular components were endoplasmic reticulum lumen, as endoplasmic reticulum lumen played a great part in protein synthesis (Figures 4D–F). KEGG pathway items suggested that *PLAU* along with its associated genes are most involved in the focal adhesion process (Figure 4G).

### Genetic Mutation, Pathway and Drug Sensitivity Analysis of Crucial Correlated Genes

*PLAU* and the top 4 correlated genes, *C10orf55*, *ITGA5*, *SERPINE1*, and *TNFRSF12A*, were chosen as crucial mediators during HNSCC progress for further analysis. As genetic alteration analysis showed, 64% of HNSCC samples exhibited *SERPINE1* genetic alteration, along with 27% samples showing *ITGA5* and *PLAU* genetic alterations respectively. Among them, missense mutation was the most popular form of genetic mutations (Supplementary Figure 1B). The distribution of variants in HNSCC samples was described subsequently. SNP acted as the main form of variant types, in which C to T alteration counted the most (Supplementary Figure 1C).

Furthermore, we investigated the relationships between these genes and carcinogenesis-related pathways. In the perspective of *PLAU* as well as its most correlated genes, the EMT process was the most enriched pathway, further identifying the role of *PLAU* in tumor invasion and metastasis (Figure 4H).

Drug sensitivity analysis indicated that low *ITGA5* showed its resistance to 14 types of drugs and small molecules consistently, while a high abundance of *TNFRSF12A* was resistant to multiple types of drugs. On the other hand, the relationship between the expression level of *PLAU*, *SERPINE1*, and drug resistance differed among drugs (Supplementary Figure 2). Importantly, high PLAU is sensitive to bleomycin and docetaxel in HNSCC. To validated the drug-sensitive data, we detected the effect of downregulating PLAU in bleomycin and docetaxel treated CAL-27 cells respectively. Suppressing PLAU led to tumor cells resistant to bleomycin and docetaxel (Supplementary Figure 3), which potentially helps the drug selection in future treatment.

### Worse Clinical Outcomes of PLAU Associated Genes in HNSCC

**FIGURE 5:**
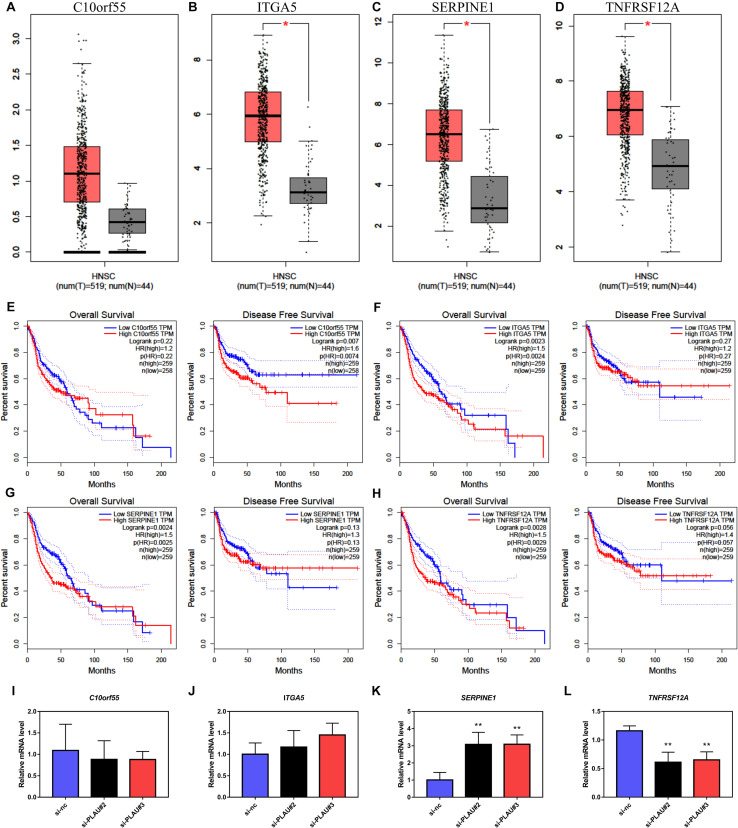
The expression levels and prognostic value of co-expressed genes in HNSCC. **(A–D)** The mRNA levels of *C10orf55*, *ITGA5*, *SERPINE1*, and *TNFRSF12A* in HNSCC and paired normal samples. **(E–H)** Prognostic role of C10orf55, ITGA5, SERPINE1, and TNFRSF12A in HNSCC. **(I–L)** The mRNA levels of *C10orf55*, *ITGA5*, *SERPINE1*, and *TNFRSF12A* in Cal27 cell lines under different si-RNA treatment.

Consequently, we presented the expression results of *C10orf55*, *ITGA5*, *SERPINE1*, and *TNFRSF12A* in HNSCC and normal samples from the TCGA database (Figures 5A–D). Even though the expression of *C10orf55* had no statistical difference between normal and tumor tissues, the expression profiles of these four genes were consistent with *PLAU*. Then the prognosis of them in HNSCC patients was also established by GEPIA (Figures 5E–H). As the results showed, HNSCC patients with lower *ITGA5*, *SERPINE1*, and *TNFRSF12A* expression had a better OS rate while there was no connection between their expression levels and DFS. However, patients with high *C10orf55* expression were correlated with poor DFS. Besides, the expression of co-expressed *C10orf55*, *ITGA5*, *SERPINE1*, and *TNFRSF12A* in Cal27 cells was detected by RT-PCR when PLAU has been downregulated. The results showed that only *TNFRSF12A* significantly decreased after downregulating PLAU, which implies TNFRSF12A is downstream of PLAU (Figures 5I–L). These results demonstrated that PLAU, as well as PLAU-associated genes, can be the potential prognostic markers of HNSCC patients.

### Lower PLAU Inhibits the Proliferation and EMT of HNSCC

**FIGURE 6:**
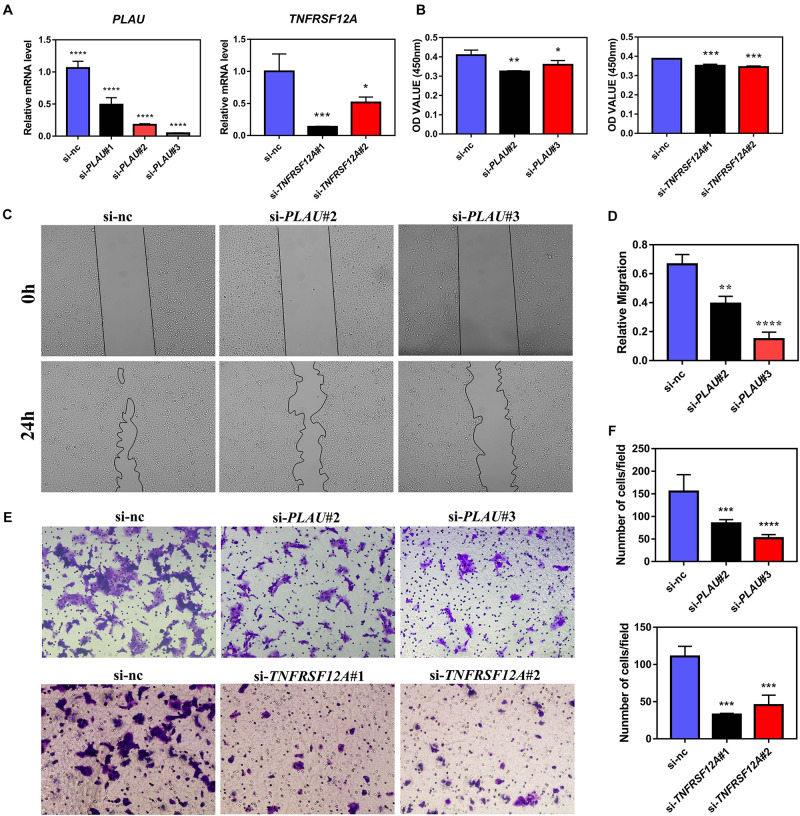
PLAU and TNFRSF12A regulate proliferation and migration *in vitro*. **(A)** Quantitative RT-PCR analysis of *PLAU* and *TNFRSF12A* expression. **(B)** Analysis of CCK8 assay. **(C,D)** Representative images and analysis of wound-healing assay. **(E,F)** Representative images and analysis of transwell assay.

**FIGURE 7:**
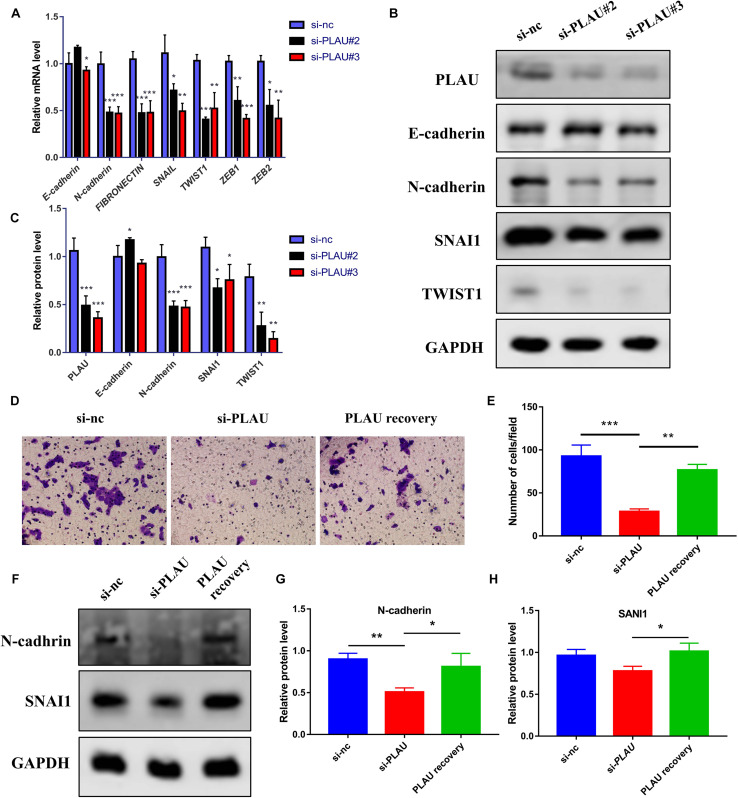
Loss of PLAU suppresses EMT process in CAL-27 cells. **(A)** Quantitative RT-PCR analysis of EMT-related markers in CAL27 cells under PLAU knockdown. **(B,C)** Western blot analysis of EMT-related proteins in CAL27 cells under PLAU knockdown. **(D,E)** Representative images and analysis of transwell assay. **(F–H)** Quantitative western blot analysis of N-cadherin and SNAI1 expression. *p < 0.05; **p < 0.01; ***p < 0.001.

Next, we evaluated the potential function of PLAU and associated genes in HNSCC cells. The PLAU-siRNA and TNFRSF12A-siRNA were transfected into the CAL27 cell line respectively and the transfected efficiency was validated by qRT-PCR (Figure 6A). Both silencings of *PLAU* and *TNFRSF12A* suppressed the proliferation of CAL-27 cells (Figure 6B). The *in vitro* experiments revealed that depletion of PLAU inhibited the migration of CAL27 cells (Figures 6C–F). To further elucidate the involvement of PLAU in the EMT pathway, we detected EMT-related markers. QRT-PCR demonstrated that *E-cadherin* was increased and *N-cadherin*, *Fibronectin*, *SNAIL*, *TWIST1*, *TWIST2*, *ZEB1*, *ZEB2* were decreased in PLAU depletion CAL27 cell line (Figure 7A). The protein levels of N-cadherin, SNAIL, TWIST1 were also downregulated in the si-PLAU group (Figures 7B,C) what’s more, recovery of PLAU would increase migration rate and N-cadherin levels while decrease the SNAI1 levels in CAL-27 cells (Figures 7D–H).

### PLAU Inhibition Suppresses the Growth and EMT of HNSCC *in vivo*

**FIGURE 8:**
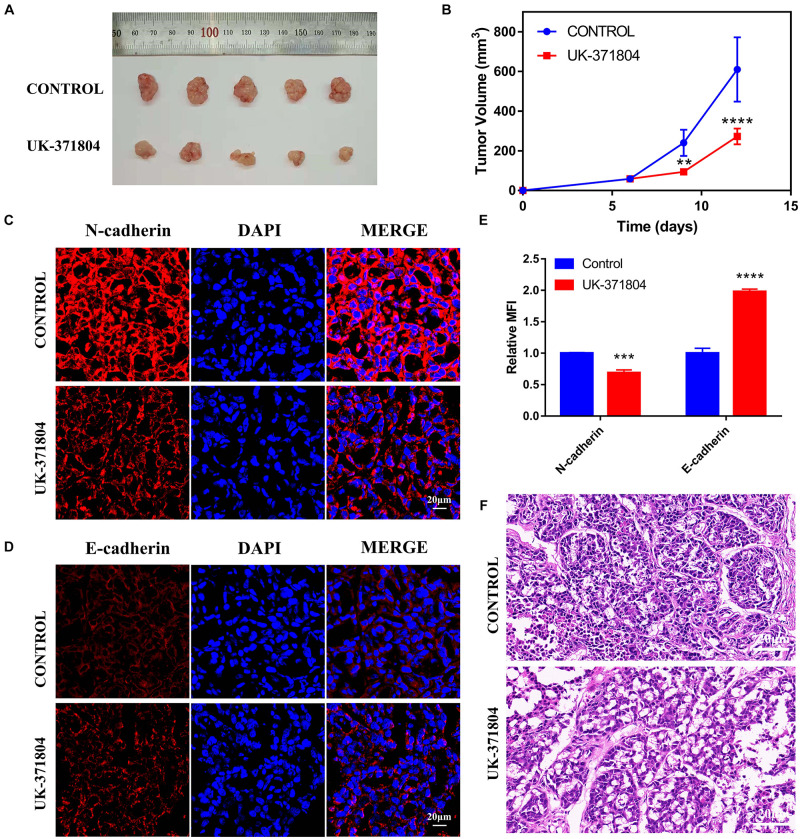
Loss of PLAU suppresses EMT and tumor growth. **(A,B)** Representative images of xenograft tumors and analysis of xenograft tumor volumes. **(C–E)** Immunofluorescent staining of N-cadherin and E-cadherin in xenograft tumors. *p < 0.05; **p < 0.01; ***p < 0.001; ****p < 0.0001. **(F)** Images of H&E staining for xenograft tumors.

To further reveal the function of PLAU on HNSCC progression *in vivo*, we construct xenografted tumor model and the mice were divided into the control group and UK-371804 (uPA inhibitor) treatment group. The UK-371804-treated group developed smaller tumor compared to the control group (Figures 8A,B). Consistent with these results, the levels of N-cadherin decreased and E-cadherin increased in UK-371804-treated group (Figures 8C–E). What’s more, the H&E staining show that the cancer cell density was lower in UK-371804-treated group (Figure 8F). Thus, intratumoral injection of UK-371804 can inhibit the progression of HNSCC.

## Discussion

In this article, the expression level of PLAU was identified to increase in HNSCC among multiple Oncomine and TCGA datasets, suggesting its important oncogenic role in HNSCC. Subsequent immunohistologic staining results validated the upregulation of PLAU in OSCC tissues. Importantly, the PLAU expression level was significantly associated with tumor grades. The prognostic analysis results agree with the results of Li et al. (2021) who have constructed the cox regression model and validated the survival results from the GEO database. Multiple types of alterations, including mRNA upregulation, amplification, and missense mutation, could explain the overexpression of PLAU to a great extent. The significant prognostic value of PLAU in HNSCC ensures its role in tumor progression. The four associated genes identified in this study, TNFRSF12A was validated to be regulated by PLAU and TNFRSF12A downregulation restrains proliferation and migration of CAL-27 cells. These results implied that the PLAU-induced EMT process may need TNFRSF12A in HNSCC. Considering that TNFRSF12A plays an important role in immune with TWEAK (Burkly et al., 2011), we also explore Expression of PLAU showed a positive relationship with macrophage while a negative relationship with CD8^+^T cell infiltration in our study (Supplementary Figure 4). It has been proved macrophage and CD8^+^T cells act as pro-tumor and anti-tumor roles respectively. Thus, PLAU might promote tumor development by posing a pro-tumor immune microenvironment in HNSCC. However, the positive correlation between PLAU and CD4^+^T cell needs further study, as two subtypes of CD4^+^T cell, Th1 and Th2 cell, act opposite roles in carcinogenesis (Chandwaskar and Awasthi, 2019). The landscape of immunity influenced by PLAU deserved further exploration, as tumor immune response was regarded as a more and more important and complex part of HNSCC progress. Also, lots of genes were identified as co-expression profiles with PLAU. These co-expression genes construct a complex interacting network, together contributing to the progress of HNSCC. As known, PLAU affects the migration and invasion of the tumor (Blasi and Sidenius, 2010). The enrichment analysis of these associated genes also showed variations of many biological processes associated with cell adhesion and extracellular matrix. In consideration that the EMT process was significantly enriched by PLAU and its co-expression genes, it is expected that PLAU might play a role in tumor invasion. Drug sensitivity analysis of PLAU and its co-expression genes suggested that PLAU expression level could guide medical strategy for HNSCC.

Accumulating bioinformatic studies indicate the crucial role of PLAU in cancer development. In gastric cancer, PLAU could act together with its co-expression gene to predict a worse survival status (Ai et al., 2020). Identification of PLAU as hub gene in pancreatic cancer through screening multiple datasets has also been completed recently (Chen Q. et al., 2019). In the perspective of HNSCC, an increase of PLAU was also observed by multiple bioinformatic analyses (Zhao et al., 2018; Yang et al., 2019). These results were just consistent with our observation, indicating that PLAU might be significant a pan-cancer oncogene. As is widely known, changes of cell-cell binding and EMT process were linked to cell migration both in epidermal cell and epithelial cell, which might lead to invasion and metastasis during tumor development (Xu et al., 2017; Aiello et al., 2018). PLAU was shown to participate in the migration and invasion of glioblastoma (Bernhart et al., 2013). In bladder cancer cells, stabilization of PLAU mRNA level might also contribute to tumor metastasis (Chen Z. et al., 2019). Considering that abnormality of cell adhesion, extracellular matrix, as well as enhancement of EMT process, were observed in our study by enrichment analysis, it might be speculated that upregulation of PLAU might promote tumor migration and invasion in HNSCC and have a clinical correlation with LN metastasis and distal metastasis. Importantly, PLAU was recommended for use as an indicator in breast cancer (Annecke et al., 2008; Sudol, 2011). We further explored the biological role of PLAU *in vitro* by manipulating the expression of PLAU. Our results demonstrated that depletion of PLAU could suppress the migration and invasion of OSCC cell line. Moreover, downregulation of PLAU reduced the EMT-related genes, indicating PLAU could induce the EMT process in the OSCC cell line. Besides, the expression and survival analysis of the top 4 associated genes of PLAU was conducted, which indicated that all these four genes and PLAU had a great predictive role in HNSCC. Drug resistance is an important factor influencing tumor prognosis (Adamska et al., 2018). Proper medical strategies were necessary for clinical treatment, which was hard to realize due to patient heterogeneity (Dagogo-Jack and Shaw, 2018). Due to drug sensitivity analysis, the association between drug sensitivity and PLAU as well as its co-expression genes was exhibited in our study. This result possesses huge clinical value, as a custom-tailored medical strategy in HNSCC could be figured out on the dependence of PLAU expression level.

Taken together, PLAU might act as an oncogene and effective biomarker in HNSCC. Enhancement of cell mobility and migration might account for its tumorigenesis. Indeed, we found that PLAU could regulate the EMT signal pathway *in vitro*. Adjustment of medical treatment based on PLAU expression might contribute to a better clinical outcome of HNSCC patients. The landscape of immunity influenced by PLAU deserved further exploration, as tumor immune response was regarded as a more and more important and complex part of HNSCC progress. *In silico* study combined experiments demonstrated that PLAU regulates the proliferation and migration via the EMT process in HNSCC.

## Data Availability Statement

The original contributions presented in the study are included in the article/Supplementary Material, further inquiries can be directed to the corresponding author/s.

## Ethics Statement

The studies involving human participants were reviewed and approved by the Institutional Research Ethics Committee of Tongji Medical College (Wuhan, China). The patients/participants provided their written informed consent to participate in this study. The animal study was reviewed and approved by Institutional Animal Care and Use Committe of Tongji Medical College.

## Author Contributions

GC and JS performed the most analysis, experiments, and writing the manuscript. SY and MX helped to revised the manuscript. QT and LC designed this study and revised the manuscript. All authors contributed to the article and approved the submitted version.

## Conflict of Interest

The authors declare that the research was conducted in the absence of any commercial or financial relationships that could be construed as a potential conflict of interest.

## Footnotes

1 www.oncomine.org

2 http://ualcan.path.uab.edu

3 https://www.cbioportal.org

4 http://gepia.cancer-pku.cn/

5 http://www.linkedomics.org

6 http://bioinfo.life.hust.edu.cn/web/GSCALite/

## Supplementary Material

Supplementary Figure 1 The single nucleotide variation (SNV) analysis of hub genes in HNSCC. **(A)** The Pearson correlation of PLAU expression with top 4 correlated genes, C10orf55, ITGA5, SERPINE1 and TNFRSF12A. **(B)** The SNV frequency and mutated types of hub genes in HNSCC. **(C)** The overview of the variation distribution of mutated genes and the classification of SNV types.

Supplementary Figure 2 Drug sensitivity analysis from Genomics of Drug Sensitivity in Cancer (GDSC) data (GSCALite). The Spearman correlation of the genes expression and drug, The positive correlation indicated that high expression is resistant to the drug, vise verse.

Supplementary Figure 3 Analysis of drug sensitivity of PLAU for bleomycin and docetaxel. ^∗^*p* < 0.05; ^∗∗^*p* < 0.01; ^∗∗∗^*p* < 0.001.

Supplementary Figure 4 Correlations between *PLAU* expression and immune infiltration level in HNSCC (Timer). **(A)** The correlation between the abundance of immune cells and the level of *PLAU*. **(B)** The correlations between *PLAU* copy number variation (CNV) and infiltration level of immune cells. ^∗^*p* < 0.05; ^∗∗^*p* < 0.01; ^∗∗∗^*p* < 0.001.

Supplementary Figure 5 Recovery of PLAU would increase N-cadherin levels and SNAI1 of CAL-27 cells. **(A)** The replicated western blots images of EMT-related proteins in CAL27 cells. **(B)** The replicated western blots images of N-cadherin and SNAI1 expression in CAL27 cells.

Supplementary Table 1 Specific si-RNA used in this study.

Supplementary Table 2 Genes and primers for PCR.
