# Supplementary figures and images for: PLAU Promotes Cell Proliferation and Epithelial-Mesenchymal Transition in Head and Neck Squamous Cell Carcinoma
Source: Front Genet. 2021 May 20;12:651882. doi: 10.3389/fgene.2021.651882 (PMC8173099; doi:10.3389/fgene.2021.651882)

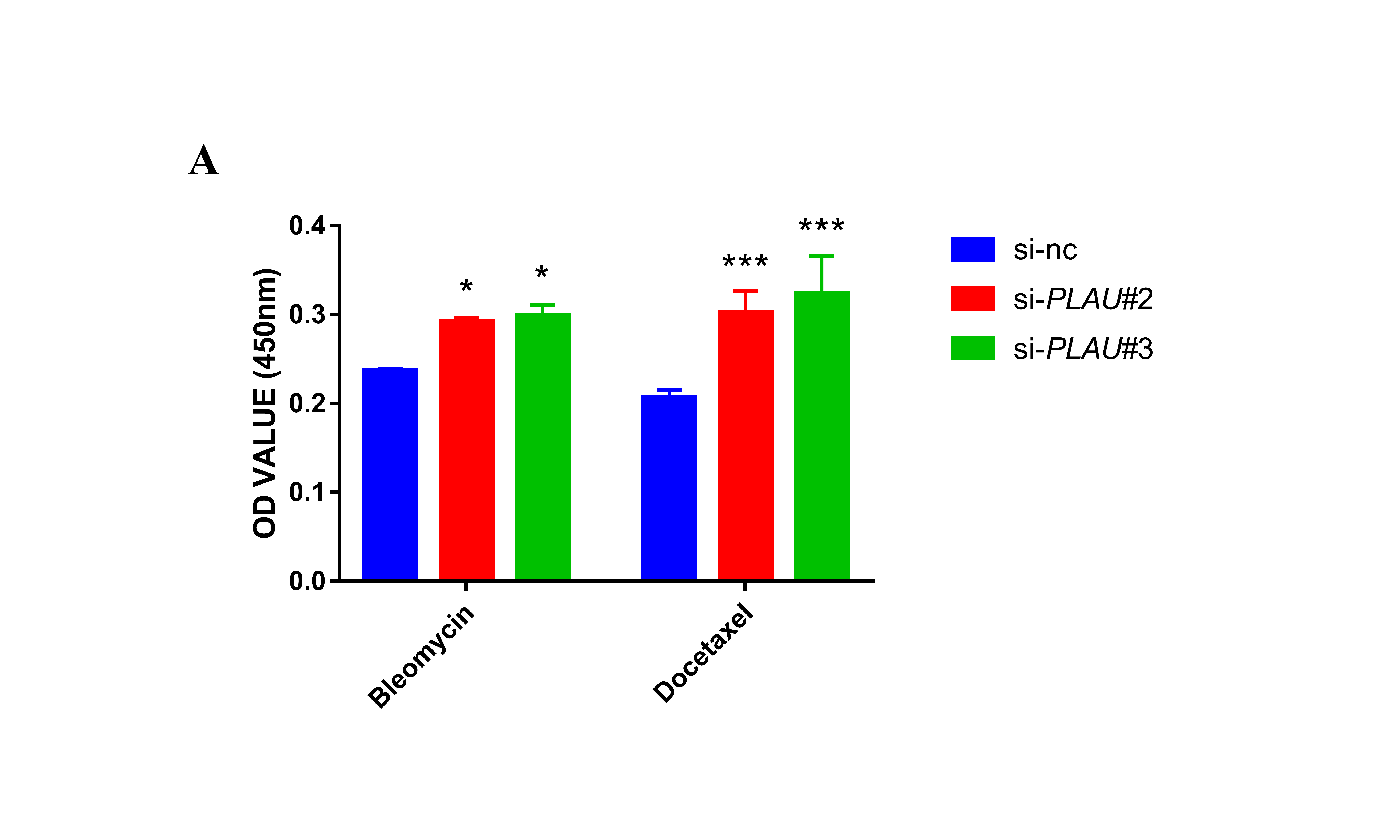

Supplement: Supplementary Figure 3 — Analysis of drug sensitivity of PLAU for bleomycin and docetaxel. ∗p < 0.05; ∗∗p < 0.01; ∗∗∗p < 0.001. [file Image_3.TIF]
