# Supplementary material for: PLAU Promotes Cell Proliferation and Epithelial-Mesenchymal Transition in Head and Neck Squamous Cell Carcinoma
Source: Front Genet. 2021 May 20;12:651882. doi: 10.3389/fgene.2021.651882 (PMC8173099; doi:10.3389/fgene.2021.651882)
Supplement: Supplementary Table 1 — Specific si-RNA used in this study. [file Table_1.DOCX]

| si-RNA | Sequences |
| --- | --- |
| PLAU-Homo-223 | 5′-GUUCCAUCGAACUGUGACUTT-3′ (sense)  5′-AGUCACAGUUCGAUGGAACTT-3′ (antisense) |
| PLAU-Homo-816 | 5′-CUGCUUCAUUGAUUACCCATT-3′ (sense)  5′-UGGGUAAUCAAUGAAGCAGTT-3′ (antisense) |
| PLAU-Homo-1303 | 5′-CGCAUGACUUUGACUGGAATT-3′ (sense)  5′-UUCCAGUCAAAGU CAUGCGTT-3′ (antisense) |
| TNFRSF12A-Homo-400 | 5′-GCAGGAGAGAGAAGUUCACTT-3′ (sense)  5′-GUGAACUUCUCUCUCCUGCTT-3′ (antisense) |
| TNFRSF12A-Homo-719 | 5′-CACUGACUAAGGAACUGCATT-3′ (sense)  5′-UGCAGUUCCUUAGUCAGUGTT-3′ (antisense) |

**Supplementary Table 1. Specific si-RNA used in this study.**
