# Supplementary material for: PLAU Promotes Cell Proliferation and Epithelial-Mesenchymal Transition in Head and Neck Squamous Cell Carcinoma
Source: Front Genet. 2021 May 20;12:651882. doi: 10.3389/fgene.2021.651882 (PMC8173099; doi:10.3389/fgene.2021.651882)
Supplement: Supplementary Table 2 — Genes and primers for PCR. [file Table_2.DOCX]

| Genes | Primers |
| --- | --- |
| *PLAU* | F-TAACGATCCCCAGTTTGGCAC  R-GGTCAGCAGCACACAGCATTT |
| *E-cadherin* | F- ATTTTTCCCTCGACACCCGAT  R- TCCCAGGCGTAGACCAAGA |
| *N-cadherin* | F- AGCCAACCTTAACTGAGGAGT  R- GGCAAGTTGATTGGAGGGATG |
| *Fibronectin* | F- AGGAAGCCGAGGTTTTAACTG  R- AGGACGCTCATAAGTGTCACC |
| *SNAI1* | F- TCGGAAGCCTAACTACAGCGA  R- AGATGAGCATTGGCAGCGAG |
| *TWIST1* | F- GTCCGCAGTCTTACGAGGAG  R- GCTTGAGGGTCTGAATCTTGCT |
| *ZEB1* | F- GATGATGAAGCGAG TCAGATGC  R- ACAGCAGTGTCTTGTTGTTGT |
| *ZEB2* | F- GGAGACGAGTCCAGCTAGTGT  R- CCACTCCACCCTCCCTTATTTC |
| *C10orf55* | F- ATTCGGGAGGAGGCTTCATCA  R- TGAGAACTAGATACGAACAGGGT |
| *ITGA5* | F- GGCTTCAACTTAGACGCGGAG  R- TGGCTGGTATTAGCCTTGGGT |
| *SERPINE1* | F- ACCGCAACGTGGTTTTCTCA  R- TTGAATCCCATAGCTGCTTGAAT |
| *TNFRSF12A* | F- AGAGAGAAGTTCACCACCCCCA  R- AATGAATGATGAGTGGGCGAGC |
| *GAPDH* | F- ACTTTGGTATCGTGGAAGGACTCAT  R- GTTTTTCTAGACGGCAGGTCAGG |

**Supplementary Table 2. Genes and primers for PCR.**
